# Shared and divergent patterns in narrative skills: comparing English monolingual and Japanese–English bilingual children

**DOI:** 10.3389/fpsyg.2026.1747702

**Published:** 2026-01-21

**Authors:** Richy Lewis Hayes, Pui Fong Kan

**Affiliations:** Department of Speech, Language, and Hearing Sciences, University of Colorado, Boulder, CO, United States

**Keywords:** bilinguals, Japanese, macrostructure, Multilingual Assessment Instrument of Narratives, story grammar

## Abstract

Narrative production requires coordinating lexical, grammatical, and discourse skills. For bilingual children, these abilities develop across two languages, influencing how resources are used during storytelling. This study compared Japanese–English bilingual and English monolingual children’s narrative microstructure and macrostructure. Twenty-eight children in each group (ages 3–8, matched for age and sex) completed the English version of the Multilingual Assessment Instrument for Narratives (MAIN). Measures included mean length of utterance in morphemes (MLUm), number of different words (NDW), subordination index (SI), fragments, story structure (SS), structural complexity (SC), internal state terms (IST), and comprehension, as well as which MAIN episode was told with the most complexity. All micro- and macrostructure measures increased with age except SC. Group differences appeared only in NDW. Age × group interactions for MLUm, SI, and NDW showed bilingual preschoolers had the lowest scores, while bilingual school-age children produced the most fragments. Age and language groups did not differ in which episode they told most complexly: all participants produced the most complex retellings for episode one and the least for episode two. Overall, microstructure was more sensitive to language experience, whereas macrostructure remained relatively stable across groups. Older children outperformed younger children, and episodic complexity patterns were consistent across age and language.

## 1 Introduction

Narrative skills are essential for the development of reading (Lynch et al., 2008; Paris and Paris, 2003), and oral language skills, such as vocabulary, grammar knowledge, and discourse structure, are strong predictors of reading skills (Miller et al., 2006; Suggate et al., 2018). For bilingual children, adequate development of the first language (L1), or the heritage language (HL), is an essential prerequisite for the reading development of the second language (L2), or the majority language (ML) (Cummins, 1979a). Early identification of language difficulties is essential for providing necessary supports for academic success; however, many norm-referenced tests (e.g., Clinical Evaluation of Language Fundamentals-Fifth Edition, Test of Narrative Language-Second Edition, etc.) are developed for monolingual English speakers, making it challenging for educators and speech-language pathologists (SLPs) to identify bilingual children with a language disorder. Research has shown that narrative tasks can be an ecologically valid way of identifying language disorders in monolingual children (Auza et al., 2018; Botting, 2002; Liles et al., 1995; Mäkinen et al., 2014; To et al., 2010) and bilingual children (Altman et al., 2016; Boerma et al., 2016; Bohnacker et al., 2022; Gutierrez-Clellen, 2002; Iluz-Cohen and Walters, 2012; Gibson et al., 2018; Jacobson and Walden, 2013; Kunnari and Välimaa, 2020; Peña et al., 2014; Squires et al., 2014; Tsimpli et al., 2016). To further this promising line of research, more studies need to be done in more languages and with participants from diverse cultural backgrounds. Much of the research has focused on Spanish-English bilinguals, and few have examined Japanese-English bilinguals. This study aims to investigate the narrative development in typically developing preschool to early school-age simultaneous Japanese-English bilingual children and compare them to age-matched English monolingual children. This comparison is significant, particularly considering a Systems Framework of bilingualism (Titone and Tiv, 2023), which states that multilingualism requires moving beyond a purely individual-centric view, and embracing the profound influence of social and environmental factors on language development. The bilinguals in the current study are not only acquiring English in academic settings, but they also attend a Japanese language and culture school, which may impact their narrative skills in a unique way, given the distinct typological differences between Japanese and English.

### 1.1 The development of narrative language skills

Monolingual preschoolers (ages 3–5) typically produce simple event descriptions, but by school age (ages 6–9) their narratives become more coherent and include story grammar elements such as scene-setting details, connectives, internal states, causal links, and evaluative comments (Berman, 1988; Bishop and Donlan, 2005; Heilmann et al., 2010; Liles, 1993; Peterson and McCabe, 1983; Poulsen et al., 1979; Stein and Glenn, 1979; van Dijk, 1976a; Westby, 2012). Narrative comprehension also improves steadily, with children showing better understanding of aurally presented stories between ages 3–7 (Curenton, 2011; Lindgren et al., 2023). Narrative discourse is commonly analyzed at the microstructure and macrostructure levels (Justice et al., 2006). Microstructure focuses on language-specific features such as syntactic complexity and lexical diversity (Košutar et al., 2022), often measured through MLUm and NDW. Macrostructure, or story grammar, examines how utterances are organized into coherent narratives (e.g., Peterson and McCabe, 1983; Stein and Glenn, 1979). This study used the Multilingual Assessment Instrument of Narratives (MAIN; Gagarina et al., 2019) to assess macrostructure, including story structure, complexity, and internal state terms.

The central question of this study is how bilingual children’s development compares to monolingual patterns when two languages are present. Several factors may influence bilinguals’ narrative development. Cultural differences in storytelling shape narrative style, and bilingual children must navigate multiple sets of practices across home, school, and peer contexts. These styles can also interact across languages—for instance, a child may apply Japanese narrative conventions when telling a story in English, or use English conventions when producing Japanese narratives (Gorman et al., 2011).

Second, bilingual children divide their language experience across two systems, resulting in less input per language than monolinguals receive. Some researchers suggest that about 60% of monolingual-level input is needed for “typical” development (Cattani et al., 2014), and reduced input has implications for how bilinguals acquire and use their languages (Daskalaki et al., 2024; De Houwer, 2007; Gathercole and Thomas, 2009). Longitudinal evidence highlights the importance of input timing and amount: Spanish–English bilingual children’s English macrostructure grew from preschool into the school years, with earlier English exposure linked to stronger initial skills, while greater home Spanish exposure predicted faster later growth (Bitetti and Hammer, 2021).

Third, vocabulary knowledge represents another critical factor influencing bilingual children’s narrative development. Research showed that when assessed in only one of their languages, bilingual children tend to obtain lower vocabulary scores than their monolingual peers (Bialystok et al., 2010; Sheng et al., 2011). However, bilingual children’s conceptual vocabulary, which is the total number of independent concepts distributed across both languages (Kan and Kohnert, 2005), is equivalent in size to monolingual children of the same age. Additionally, the intralanguage vocabulary differences may impact narrative language development, as vocabulary has been shown to be strongly correlated with narrative competence (Altman et al., 2016; Chan et al., 2023; Hammer et al., 2012; Heilmann et al., 2010; Iluz-Cohen and Walters, 2012; Košutar et al., 2022).

Fourth, research on dual language development suggests bilingual children often outperform monolinguals in metalinguistic awareness, particularly on tasks requiring high cognitive control (Bialystok, 2001). This advantage may influence how bilinguals develop narrative discourse skills involving causality and theory of mind (Gagarina et al., 2019). Below, we review research comparing bilingual and monolingual children’s narrative microstructure and macrostructure.

### 1.2 Microstructure in bilingual and monolingual children

Microstructure (e.g., lexical diversity, morphosyntactic marking) is said to depend on language experience (e.g., Rodina, 2017) and varies across languages. For bilingual children, this often appears as differences in vocabulary surface forms, such as knowing a phonological form in only one language. As they age, bilingual children acquire more translation equivalents (Kan and Kohnert, 2005; Sheng et al., 2011), so vocabulary differences may appear in their narratives relative to monolinguals. Boerma et al. (2016) found that both typically developing and language-impaired bilingual children scored significantly lower on vocabulary tests than typically developing monolinguals. Hipfner-Boucher et al. (2015) similarly reported that only English learners who spoke mostly the home language (HL) scored lower in lexical diversity than monolinguals and English learners dominant in the majority language (ML). This could be explained differing amounts of input to the academic language, or the ML. From a broader theoretical perspective, such reduced exposure may delay the development of cognitive academic language proficiency (CALP), which depends on cumulative language experience, first-language proficiency, and age, and which emerges more slowly than basic interpersonal communicative skills (BICS; Cummins, 1979b). Additionally, Narrative tasks draw heavily on CALP, because they require decontextualized language, cohesion, causality, internal state descriptions, and abstract vocabulary. Kunnari et al. (2016) also observed differences between Finnish monolingual and Finnish–Swedish bilingual children: both groups produced similar word counts, but monolinguals produced more utterances. Kubota et al. (2022) found that token–type ratio in Japanese–English bilinguals depended on continued English exposure after returning to Japan. Finally, Dosi et al. (2024) observed early greater literacy exposure predicted greater noun diversity in Greek-Turkish bilingual children’s Greek. Collectively, these studies show that microstructure—especially vocabulary—is shaped by language experience, and increased exposure supports higher lexical diversity in bilinguals’ narratives.

In addition to vocabulary, small variations in morphosyntax can be seen in the development of narrative discourse in bilinguals and monolinguals of different language backgrounds. Slobin (1996) compared monolingual English and Spanish speakers and found differences in verb use even when macrostructure remained consistent – monolingual English children tended to use a more manner verbs, while monolingual Spanish children used more path verbs. Otwinowska et al. (2020) found that Polish-English bilinguals’ Polish narratives showed an overuse of function words, particularly pronouns. Rodina (2017) observed significantly more errors in morphosyntax in bilingual Norwegian-Russian preschoolers versus monolingual Norwegian and monolingual Russian preschoolers. Rezzonico et al. (2016) observed that Cantonese-English preschoolers tended to use simple syntax. Analysis of the English grammar errors showed that the source of agrammaticality was primarily verbal morphology omissions in obligatory contexts or incomplete BE plus -ing forms. Finally, Mishina-Mori et al. (2024) compared Japanese-English bilingual children with Japanese monolingual children, and observed cross-linguistic influence from English to Japanese in how the bilinguals reintroduced referents. They found that the bilingual children used more noun phrases, compared to the monolingual children who more often dropped the pronoun, to reintroduce characters in a story retell. These findings parallel similar findings from Chen and Lei (2013), who found that for referent re-introduction in Chinese, bilingual speakers produced more definite noun phrases and dropped fewer pronouns than their monolingual peers. On the other hand, Dosi et al. (2024) found that exposure to literacy in Greek, the L2, leads to greater syntactic complexity. While subtle, the differences in information structure from language to language appear to cross linguistic boundaries. In summary, acquisition of different languages, or multiple languages, can impact not only vocabulary usage, but also morphosyntactic patterns.

### 1.3 Macrostructure in bilingual and monolingual children

Early literacy practices are important for later language and reading outcomes, specifically, parent language during book reading contains greater lexical diversity and syntactic complexity, leading to improved language and literacy skills in their children (Ece Demir-Lira et al., 2019). Some studies have found subtle differences in narrative macrostructure depending on cultural storytelling norms. One study being Gorman et al. (2011), who compared 60 first- and second-grade African American, Latino American, and Caucasian children. They found that culture influenced the children’s narrative production on a wordless picture book task. They called for a better understanding of narrative structure, creativity, and style to provide ecologically valid narrative assessment and intervention for children from diverse cultural backgrounds. Similarly, Fiestas and Peña (2004) examined Spanish–English bilingual children and found that, although overall story organization remained coherent across languages, the distribution of narrative elements differed. In Spanish, children produced proportionally more references to initiating events and character intentions, whereas in English they provided more statements related to outcomes and consequences. These findings suggest that macrostructure involves both shared cognitive frameworks for organizing narrative events and culturally mediated discourse preferences that shape how narrative meaning is developed. Thus, examining English macrostructure in Japanese–English bilingual children relative to monolingual peers provides an important opportunity to determine whether narrative organization in English reflects a shared cross-linguistic framework or whether culturally and linguistically rooted patterns from Japanese storytelling continue to shape how children structure their narratives.

In contrast to microstructure, macrostructure is generally less affected by language-specific knowledge (e.g., Andreou and Lemoni, 2020; Boerma et al., 2016; Bohnacker et al., 2022). Macrostructure refers to the higher-order organization of discourse, or story grammar (Gagarina et al., 2019). These “narrative superstructures” (van Dijk, 1980)—such as setting, complication, resolution, evaluation, and moral—are predictable elements that shape a story’s global content. Van Dijk (1980) described macrostructures as the underlying semantic “gist” that enables summarization. Episodic Analysis (Stein and Glenn, 1979) is a macrostructural framework based on the idea that stories are organized around characters’ goal-directed actions. MAIN (Gagarina et al., 2019) adapts this framework into a clinical tool for assessing micro- and macrostructure in bilinguals, though it is also useful for monolinguals (Pesco and Kay-Raining Bird, 2016). This tool has supported extensive research on narrative development.

Kunnari et al. (2016) compared Finnish–Swedish bilingual and Finnish monolingual children using the MAIN and found group differences in SS only for storytelling, not retelling. They also reported that SC—specifically episodes containing Goal statements—increased with age, though complete and abbreviated episodes were rare. Tsimpli et al. (2016) compared monolingual and bilingual Greek-speaking children, including age-matched TD and SLI groups. While SLI groups scored lower than TD groups, both bilingual groups outperformed monolinguals on story production, and the bilingual SLI group outperformed the monolingual SLI group on comprehension, suggesting a potential bilingual advantage in story grammar. Otwinowska et al. (2020) found no differences between Polish–English bilinguals and Polish monolinguals in SS, ISTs, or comprehension on the MAIN. Lipner et al. (2024) showed that age predicted macrostructure in Hebrew–English bilinguals, with older children producing more macro elements. Macrostructure correlated across languages, indicating cross-linguistic transfer. They also found that episode three was told with the greatest complexity, likely because character relationships become explicit in the final episode. Overall, these studies show that macrostructure does not differ substantially between bilinguals and monolinguals and increases linearly with age in both groups.

### 1.4 The current study

The aim of the current study is to examine shared and divergent patterns in the English narratives of Japanese-English bilingual children ages three to eight and their age-matched English monolingual peers. We focused solely on the English narratives in this study for two reasons: English is the ML and the language of instruction, so all the children in the study have academic English skills; and focusing on English allowed a one-to-one comparison of the microstructure and macrostructure between the two groups. This study parallels many previous studies that used MAIN to examine narrative macrostructure in bilingual children, and most closely resembles Kunnari et al. (2016), in that it compares the narratives of typically developing bilingual children to typically developing monolingual children. Additionally, similar to Altman et al. (2016) and Hao et al. (2019), our study also examines the narratives of bilinguals who speak typologically distant languages.

The research questions guiding our study are:

1. How do Japanese–English bilingual children differ from English monolingual peers in English microstructure (i.e., MLUm, NDW, SI, and number of fragments)?
2. How do Japanese–English bilingual children compare with monolinguals on English macrostructure (i.e., story structure, structural complexity, internal state terms, and comprehension)?
3. To what extent does age group (preschool vs. school-age)—reflecting both developmental maturation and accumulated school experience—predict English microstructure and macrostructure?
4. Does age group (preschool vs. school-age) or bilingualism (monolingual vs. bilingual) affect which of the three episodes is told with more complexity in the English narratives of Japanese-English bilingual and English monolingual children?

For research question one, we hypothesize that there may be subtle differences in all microstructure measures, as evidenced by much of the research on bilingual vocabulary differences (e.g., Bialystok et al., 2010; Kan and Kohnert, 2005), as well as more subtle differences in morphosyntax due to cross-linguistic influence from Japanese (Chen and Lei, 2013; Mishina-Mori et al., 2024). For Research Question Two, we hypothesize that Japanese–English bilingual and English monolingual children will show similar overall macrostructure scores, reflecting the relatively language-general nature of story organization. However, drawing on research showing culturally mediated variation in how narrative elements are emphasized (Fiestas and Peña, 2004; Gorman et al., 2011), we anticipate that subtle differences may emerge in the distribution or elaboration of specific macrostructural components, even when global structure remains comparable. This pattern would be consistent with the view that narrative macrostructure is a shared cognitive framework shaped by culturally influenced discourse practices. Additionally, Hayes and Kan (2025) found evidence to support the transfer of underlying lexical semantic knowledge between languages in Japanese-English bilingual children, despite a discrepancy in language ability. For research question three, we hypothesize that the school-age children will have higher microstructure and macrostructure scores than the preschool children; much of the research has shown improvements in both with age (e.g., Brown, 1973; Durán et al., 2004; Bohnacker et al., 2022; Uccelli and Páez, 2007). Finally, for research question four, we hypothesize that, like Lipner et al. (2024), episode three of the MAIN will have the most SC components for both groups, since macrostructure skills are considered relatively stable across linguistic contexts; the preschool children may show more variety in which episode they tell with greater complexity, due to nascent macrostructure competence.

## 2 Methods

### 2.1 Participants

Participants were 56 children aged three to eight (M = 73.6 months, SD = 20.1 months) attending public school in Colorado. Half of the children were Japanese-English bilingual and half were age-matched monolingual English-speaking children. The bilingual group consisted of 28 simultaneous Japanese-English bilingual children (they began acquiring both languages younger than four; Genesee and Nicoladis, 2006) who also attend the Japanese Academy of the Rockies, a Saturday-only school that teaches Japanese language, literacy, and culture. These children have at least one parent who is native speaker of Japanese (12 girls; M = 73.53 months, SD = 20.25 months). Additionally, 21 of the 28 bilingual children reported being more comfortable speaking English, four more comfortable speaking Japanese, and three equally comfortable speaking Japanese and English. The age-matched monolingual group consisted of 28 English-speaking children (12 girls; M = 73.71, SD = 20.36). They were recruited from a local children’s summer camp, within roughly the same geographical area as the Japanese Academy of the Rockies. Both groups lived in a middle- to upper middle-class area of Colorado. There was no significant difference in chronological age among the groups, *t*(54) = 0.03, *p* = 0.97. We used a caregiver questionnaire that was adapted from Cheung et al. (2018) for Japanese-English bilingual and monolingual children to query demographic information, developmental history, and language environment (see Table 1). According to the questionnaire, all participants were considered typically developing based on not having an Individualized Education Plan (IEP) and not receiving special education services. Next, maternal education was measured using an ordinal scale: “lower than high school” being 1, “GED or high school” being 2, “associate’s degree/some college” being 3, “bachelor’s degree” being 4, and “Master’s/MD/JD/PhD” being 5. The difference between the monolingual’s maternal education (M = 4.43) and the bilingual’s maternal education (M = 3.89) was nonsignificant, *z* = 1.6, *p* = 0.10. Finally, there was no significant difference between the number of hours of total language input the children received at home, *t*(54) = 0.6, *p* = 0.55. However, there was a significant difference between the number of hours of English input the children received at home, *t*(54) = 5.33; *p* = 0.001. The bilinguals received an average of 8.6 h of English input and 7 h of Japanese input (15.6 h of total language input), while the monolinguals received an average of 16.5 h of input at home.

**Table 1:** Caregiver questionnaire information for both groups (*n* = 56).

Demographic information	Monolinguals	Bilinguals
	*n*	*n*
Age		
*M*	73.7 months	73.5 months
3	2	1
4	6	8
5	7	5
6	3	3
7	5	6
8	5	5
Gender		
Female	12	12
Male	16	16
Maternal education		
Average	4.42	3.89
Weekly at-home language input		
English	16.53 h	8.63 h
Japanese	N/A	7.01 h

### 2.2 Materials and procedures

To elicit a language sample for micro and macrostructural ability, we used the Multilingual Assessment Instrument of Narratives (MAIN; Gagarina et al., 2019). The MAIN was developed to assess narrative production and comprehension skills of children from 3- to 10-years-old. It can be used as a story tell, retell, or model story. The dog and cat parallel stories were used as a story retell in this study.

The monolingual children only completed one testing session in English. Half of the children did the “Cat Story,” and the other half did the “Dog Story.” The bilingual children competed two stories, one in Japanese and one in English, at least one week apart, with sometimes longer between sessions depending on the family’s schedule. The bilingual group was tested for a larger study, thus, while the bilingual group was assessed in both languages, we focus on the English narratives in this study. The bilingual children were tested at the Japanese Academy of the Rockies, a heritage language school that is held on Saturdays. The examiners included three trained monolingual English-speaking research assistants (RAs), the PI – who is a Japanese-English bilingual speaker – and a trained native Japanese-English bilingual RA. To create an environment conducive to the monolingual mode (Grosjean, 1998), the children were tested by a different examiner for both tests, and all interaction prior to the testing, and instructions given during the testing, were done strictly in the testing language. The participants were given the option of three stories in envelopes to choose from; however, each envelope contained the same story. This was to control for the effect of shared knowledge during the presentation of the pictures (Gagarina et al., 2019). After the child chose the envelope, the examiner would display the pictures while reading the story, then prompt the child to tell the story back, “in their own words.” The order of administration and language for the bilingual children was counterbalanced.

All testing procedures were the same as the bilingual children, except all testing and interaction with the children and their families was done in English. Both groups’ narratives were recorded using OM System Olympus WS-882 Digital Voice Recorders.

### 2.3 Macrostructure measures

Four macrostructure measures were used as dependent variables: Story structure (SS), structural complexity (SC), internal state terms (IST), and story comprehension.

SS is scored on a 17-point scale, where two possible points are given for a setting statement (consisting of time and place), followed by three episodes comprised of five components (which equate to points) each: i) an internal state to initiate the goal, ii) a goal statement for a character iii) an attempt by a character, iv) an outcome of the attempt, and v) an internal state which expresses the character’s reaction.

SC was scored using a 3-point system outlined by Maviş et al. (2016). A sequence without Goal (i.e., Attempt-Outcome) would be given 1 point. An incomplete episode (single Goal, Goal-Attempt or Goal-Outcome) would be given 2 points. A complete episode (Goal-Attempt-Outcome) would be given 3 points. Each MAIN story consists of three stories, so a score of nine points (three GAO sequences) is the ceiling. The MAIN manual (Gagarina et al., 2019) scores by totaling the number of each type of sequence observed. We used a 3-point system to examine gradual developmental trends with age and compare the complexity of each episode within the story.

ISTs were totaled. Examples of ISTs from the MAIN manual include: Perceptual terms (e.g., see, hear, feel); physiological terms (e.g., thirsty, hungry, tired); consciousness terms (e.g., alive, awake, asleep); emotion terms (e.g., sad, happy, angry); mental verbs (e.g., want, think, know); linguistic verbs (e.g., say, call, shout).

Comprehension questions were scored on a scale of 1 to 10. Answers were scored based on the MAIN manual (Gagarina et al., 2019).

Finally, an aggregate score was derived by totaling all the macrostructure scores (i.e., SS, SC, IST, and comprehension). Within the current study, the aggregate score range was 13 to 47 (M = 25.91, SD = 6.94).

### 2.4 Microstructure measures

Four microstructure measures were used as dependent variables: Mean Length of Utterance in Morphemes (MLUm), Number of Different Words (NDW), Subordination Index (SI), and Fragments ([F]).

MLUm is a general index of syntactic development – as utterance length increases, syntactic complexity increases, e.g., number of semantic roles, negatives, auxiliaries, and clauses (Brown, 1973). MLUm is a reliable, global measure of syntactic complexity.

NDW is a commonly used measure of lexical diversity (Justice et al., 2006; Miller et al., 2006). Some studies have found that the lexical diversity measure “D” is a more reliable measure (Durán et al., 2004), however, Jacobson and Walden (2013) found the two to be highly correlated in both languages of Spanish-English bilinguals. Additionally, NDW is a sensitive measure of narrative productivity (Altman et al., 2016; Uccelli and Páez, 2007).

SI is a useful measure of syntactic complexity (Justice et al., 2006), measured by the ratio of the total number of clauses to the total number of C-units (Miller et al., 2019). Brown (1973) suggested that once MLUm is over an average of 4.0, it is no longer considered an accurate measure; therefore, since the age range in this study extended to eight years, SI was used as a more sophisticated measure of syntactic complexity to account for any insufficiencies in MLUm.

[F] utterances contain a succession of verbs without subjects. The subjects in these can be implied due to segmentation (Miller et al., 2019). In Japanese, personal pronouns are frequently dropped to avoid redundancy (Kaiser et al., 2013, p. 662), so the number of pro-drop utterances was noted to observe any potential crosslinguistic influence.

### 2.5 Transcription, scoring, and analysis

The children’s narratives were transcribed and analyzed using Systematic Analysis of Language Transcripts (SALT) software (Version 20; Miller et al., 2019). The audio recordings were transcribed and coded following SALT conventions by the PI and a trained RA. The transcripts were coded to mark individual words and bound morphemes to calculate MLUm and NDW. To calculate SI, the number of clauses was marked with the code “SI-[number]” to indicate the number of clauses per utterance (e.g., SI-1 for one clause, SI-2 for a main and subordinate clause, etc.). Finally, fragments were identified as well-formed utterances in an immediate sequence, where the first utterance contains the subject and the following utterances do not, but the subject can be implied (e.g., “C He got on the rock. C and fell off the rock [F].”) These utterances were marked with the code “[F]” to calculate the total number per transcript. Macrostructure was then scored following the scoring sheet provided by the MAIN, with the addition of the aggregate sum of each element from the MAIN rubric. To note which episode yielded the most GAO elements, a complexity score of 1, 2, or 3 was given to each of the three MAIN episodes based on the total number of GAO elements included in that episode. For example, if an episode included only a single G, A, or O, it was given a 1; if it included a GA, GO, or AO, it was given a 2; and if it included all three, GAO, then it was given a 3. For interrater agreement, a 10% overlap of the narratives were coded and scored for macrostructure by the PI and RA. Comparison of the SALT codes showed a 99% item agreement, and comparison of the MAIN macrostructure scoring sheets showed a 95% item agreement. Microstructure and macrostructure elements acted as dependent variables. Finally, each participant was indicated as monolingual/bilingual and preschool/school-age, which were independent variables.

## 3 Data analysis

Preliminary correlation analyses were conducted to explore trends in variables (see Table 2 for monolinguals and Table 3 for bilinguals). Age, MLUm, NDW, SI, SS, SC, IST, comprehension, and aggregate were compared to observe changes in development.

**Table 2:** Correlations for various study variables (monolingual).

Variable	1	2	3	4	5	6	7	8
1. Age	–							
2. MLUm	0.22*	–						
3. NDW	0.1	0.1	–					
4. SI	0.07	0.06	0.18*	–				
5. SS	0.18*	0.02	0.37***	0.25**	–			
6. SC	0.002	0.04	0.09	0.03	0.32**	-		
7. IST	0.14*	0.07	0.26**	0.46***	0.47***	0.05	–	
8. Comp	0.07	0.00	0.001	0.11	0.09	0.009	0.09	–
9. Aggregate	0.16*	0.05	0.3**	0.37***	0.84***	0.38***	0.71***	0.21*

*p < 0.05. **p < 0.01. ***p < 0.001. MLUm; Mean Length of Utterance in Morphemes, NDW; Number of Different Words, SI; Subordination Index, SS; Story Structure, SC; Structural Complexity, IST; Internal State Terms, Comp; Story Comprehension, Aggregate; Sum score of SS, SC, IST, & Comp.

**Table 3:** Correlations for various study variables (bilingual).

Variable	1	2	3	4	5	6	7	8
1. Age	–							
2. MLUm	0.43***	–						
3. NDW	0.46***	0.36***	–					
4. SI	0.48***	0.62***	0.38***	–				
5. SS	0.34**	0.54***	0.55***	0.49***	–			
6. SC	0.09	0.3**	0.22*	0.14	0.44***	–		
7. IST	0.29**	0.45***	0.46***	0.43***	0.85***	0.3**	–	
8. Comp	0.27**	0.34**	0.17*	0.23**	0.1	0.07	0.08	–
9. Aggregate	0.37***	0.62***	0.55***	0.5***	0.92***	0.56***	0.86***	0.24**

*p < 0.05. **p < 0.01. ***p < 0.001. MLUm, Mean Length of Utterance in Morphemes; NDW, Number of Different Words; SI, Subordination Index; SS, Story Structure; SC, Structural Complexity; IST, Internal State Terms; Comp, Story Comprehension; Aggregate, Sum score of SS, SC, IST, & Comp.

Separate 2×2 analyses of variance (ANOVAs) was used to answer research questions one through three, with Language group (monolingual or bilingual) and Age group (preschool or school-age) as independent variables, and microstructure (MLUm, NDW, SI, and [F]) and macrostructure (SS, SC, IST, comprehension, and aggregate) as dependent variables. Research question four used a linear mixed effects model with Language group, Age group, and Episode number (one, two, and three) as fixed effects, subject as a random effect, and Number of GAO Elements as the outcome.

## 4 Results

### 4.1 Descriptive statistics

Table 4 shows micro and macrostructure scores by group. Table 5 shows the frequency of each individual SS component. Table 6 shows accuracy on each type of comprehension question. The accuracy was equal for the theory of mind (ToM) 1 question, and the bilinguals’ accuracy was much higher for the ToM2 question, although not significant, *X*^2^ = (1, *N* = 56) = 1.82, *p* = 0.18. Both groups scored close to the ceiling on Goal and Internal State questions, with slightly lower accuracy on the ToM1 question, and the most significant drop in accuracy on the ToM2 question.

**Table 4:** Microstructure and macrostructure scores by group.

Group	Microstructure		SI	[F]	Macrostructure		IST	Comp	Aggregate
	MLUm	NDW			SS	SC			
Monolingual									
*M*	7.28	47.36	1.12	0.46	8.86	3.39	5.71	9.07	27.03
*SD*	1.31	10.59	0.13	0.79	2.22	1.62	2.65	1.01	5.73
*Range*	4.12–10.3	29–74	0.86–1.36	0–3	5–13	1–7	0–10	6–10	14–37
Bilingual									
*M*	7.13	39.29	1.09	0.82	8.21	3	4.54	9.04	24.79
*SD*	2.05	14.03	0.15	1.18	2.85	1.87	3.26	1.55	7.9
*Range*	2.74–12	20–71	0.67–1.38	0–4	4–16	0–9	0–13	3–10	13–47

MLUm, Mean Length of Utterance in Morphemes; NDW, Number of Different Words; SI, Subordination Index; SS, Story Structure; SC, Structural Complexity; IST, Internal State Terms; Comp, Story Comprehension; Aggregate, Sum score of SS, SC, IST, & Comp.

**Table 5:** Frequency of the individual story components in production.

Story structure component	Monolingual	Bilingual
Setting	19%	14%
Initiating Event IST	55%	52%
Goal	29%	22%
Attempt	68%	62%
Outcome	85%	88%
Reaction IST	46%	39%

IST, Internal State Term.

**Table 6:** Accuracy with different types of comprehension questions: goals (questions 1, 4, 7), internal states (questions 2, 3, 5, 6), ToM1 (questions 8, 9), ToM2 (question 10).

Comprehension question type	Monolingual	Bilingual
Goals	98%	96%
Internal States	99%	95%
ToM1	91%	91%
ToM2	**36%**	**54%**

ToM, Theory of Mind.

### 4.2 Research questions one through three: age and language group

**Figure 1:**
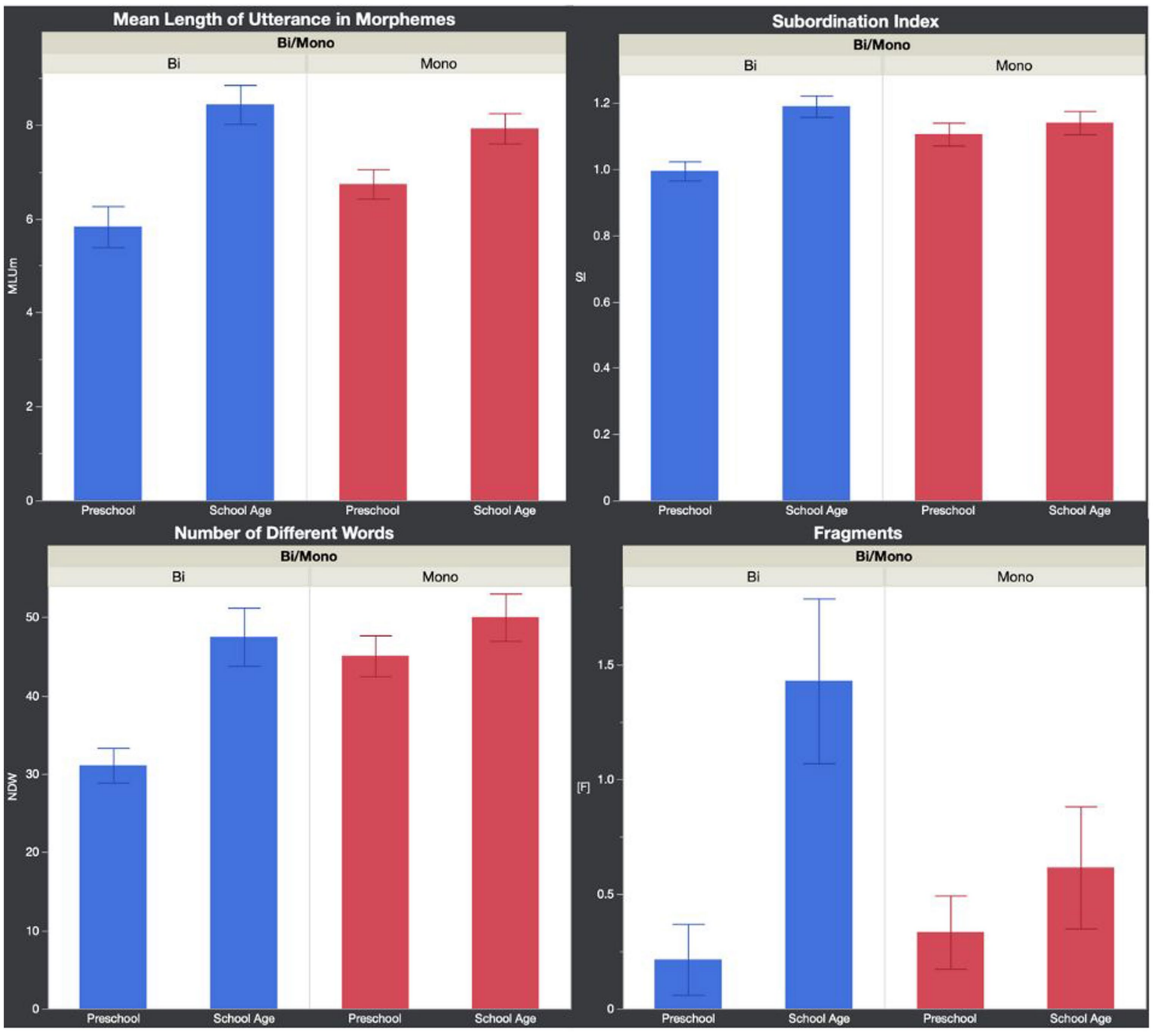
Narrative microstructure performance per group. MLUm, mean length of utterance in morphemes; SI, subordination index; NDW, number of different words; [F] = fragments (error bars show standard error).

**Figure 2:**
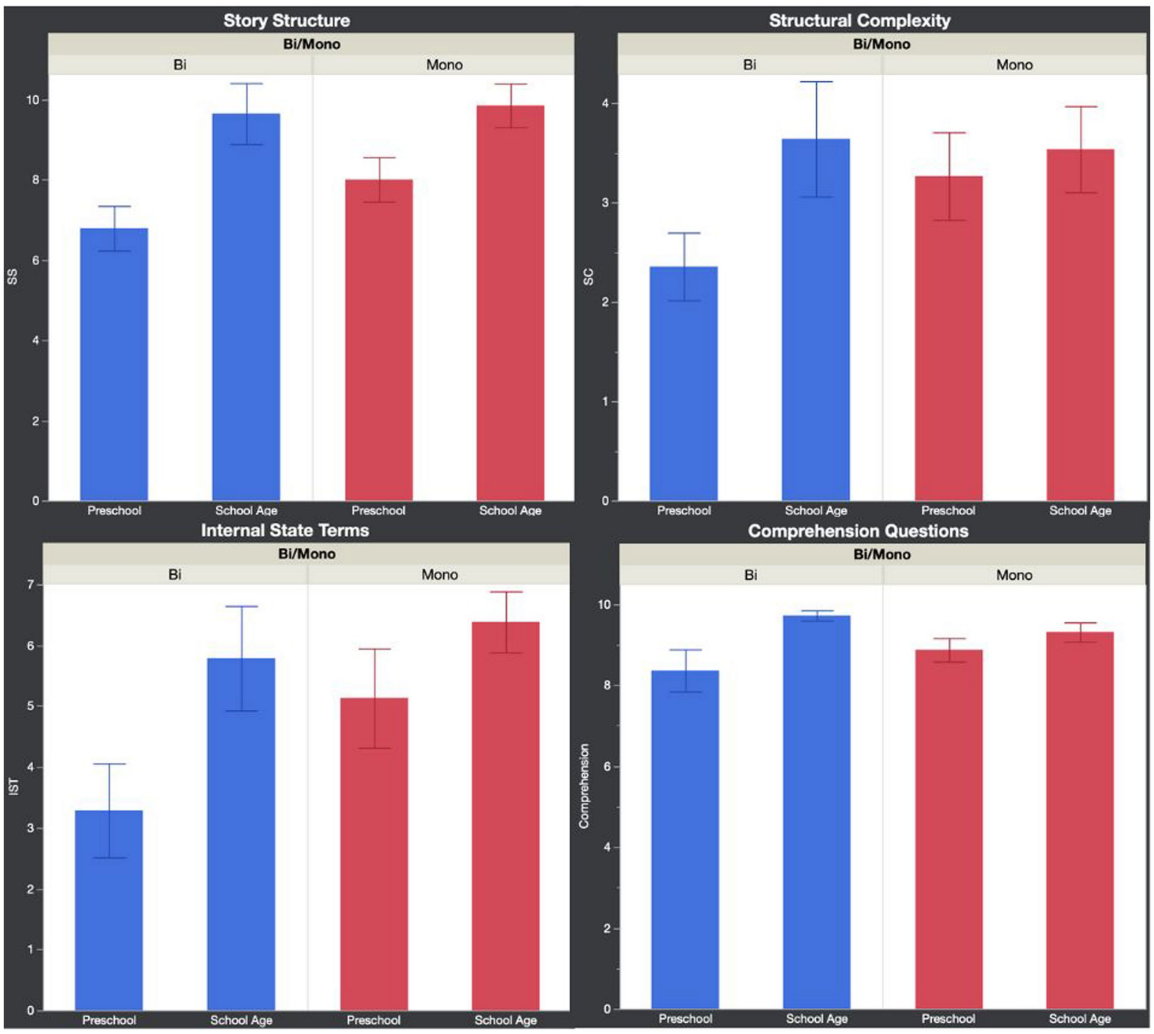
Narrative macrostructure performance per group. SS, story structure; SC, structural complexity; IST, internal state terms (error bars show standard error).

Tables 7, 8 summarize the results examining the effects of Language and Age group on English micro and macrostructure. Figure 1 shows mean microstructure performance, and Figure 2 shows mean macrostructure performance for each group.

**Table 7:** ANOVA results for language and age group: microstructure.

Variable	MLUm		NDW		SI		[F]	
	*df*	*F*	*df*	*F*	*df*	*F*	*df*	*F*
Language	3, 52	0.28	3, 52	7.86**	3, 52	0.86	3, 52	1.98
Age	3, 52	25.44***	3, 52	13.18***	3, 52	12.28**	3, 52	9.19**
Language*Age	3, 52	3.58+	3, 52	3.82+	3, 52	5.99*	3, 52	3.57+

^+^*p <* 0.1, ^∗^*p <* 0.05, ^∗∗^*p <* 0.01, ^∗∗∗^*p <* 0.001. MLUm, Mean Length of Utterance in Morphemes; NDW, Number of Different Words; SI, Subordination Index; F, Fragments.

**Table 8:** ANOVA results for language and age group: Macrostructure.

Variable	SS		SC		IST		Comprehension		Aggregate	
	*df*	*F*	*df*	*F*	*df*	*F*	*df*	*F*	*df*	*F*
Language	3, 52	1.34	3, 52	0.77	3, 52	2.57	3, 52	0.02	3, 52	2.04
Age	3, 52	14.8***	3, 52	2.89	3, 52	6.05*	3, 52	7.47**	3, 52	12.49***
Language*Age	3, 52	0.68	3, 52	1.22	3, 52	0.67	3, 52	1.94	3, 52	1.57

^+^*p <* 0.1, ^∗^*p <* 0.05, ^∗∗^*p <* 0.01, ^∗∗∗^*p <* 0.001. SS, Story Structure; SC, Structural Complexity; IST, Internal State Terms; Aggregate, Sum of SS, SC, IST, and Comprehension.

For microstructure, MLUm was significant, *F*(3, 52) = 9.75, *p <* 0.001, *R*^2^ = 0.36, with Language group not being significant, *p* = 0.60, the interaction between Language and Age group nearing significance, *p* = 0.06, and Age group being significant, *p <* 0.001. To explore the near-significant interaction, Tukey HSD post-hoc analysis revealed significant differences between bilingual preschoolers (*M* = 5.82) and bilingual school-age (*M* = 8.43), *p <* 0.001, and bilingual preschoolers (M = 5.82) and monolingual school-age (*M* = 7.92), *p <* 0.01.

NDW was significant, *F*(3, 52) = 8.19, *p <* 0.001, R^2^ = 0.32, with Language group, *p <* 0.01, and Age group, *p <* 0.001, both being significant, and the interaction between Language and Age group nearing significance, *p* = 0.06. To further explore the near-significant interaction, Tukey HSD post-hoc analysis revealed significant differences between bilingual preschoolers (*M* = 31.07) and bilingual school-age (*M* = 47.5), *p <* 0.01, bilingual preschoolers (*M* = 31.07) and monolingual preschoolers (*M* = 45.07), *p <* 0.01, and bilingual preschoolers (*M* = 31.07) and monolingual school-age (M = 50), *p <* 0.001.

SI was significant, *F*(3, 52) = 6.37, *p <* 0.001, *R*^2^ = 0.27, with Language group not being significant, *p* = 0.36, and Age group, *p <* 0.01, and the interaction between Language and Age group being significant, *p <* 0.05. To further explore the interaction, Tukey HSD post-hoc analysis revealed significant differences between bilingual preschoolers (*M* = 0.99) and bilingual school-age (*M* = 1.19), *p <* 0.001, and bilingual preschoolers (M = 0.99) and monolinguals school-age (*M* = 1.14), *p <* 0.05.

Fragments were significant, *F*(3, 52) = 4.97, *p <* 0.01, *R*^2^ = 0.22, with Language group not being significant, *p* = 0.17, the interaction between Language and Age group nearing significance, *p* = 0.06 and age group being significant, *p <* 0.01. To further explore the near-significant interaction, Tukey HSD post-hoc analysis revealed significant differences between bilingual preschoolers (*M* = 0.21) and bilingual school-age (*M* = 1.43), *p <* 0.01, and monolingual preschoolers (*M* = 0.33) and bilingual school-age (*M* = 1.43), *p <* 0.05.

For macrostructure, SS was significant, *F*(3, 52) = 5.54, *p <* 0.01, *R*^2^ = 0.24, with Language group, *p* = 0.25, and the interaction between Language and Age group, *p* = 0.41, not being significant, and Age group being significant, *p <* 0.001.

SC was not significant, *F*(3, 52) = 1.62, *p* = 0.2, *R*^2^ = 0.09, and none of the variables reached significance.

ISTs were significant, *F*(3, 52) = 3.04, *p <* 0.05, *R*^2^ = 0.15, with Language group, *p* = 0.11, and the interaction between Language and Age group, *p* = 0.42, not being significant, and Age group being significant, *p <* 0.05.

Comprehension was significant, *F*(3, 52) = 3.15, *p <* 0.05, *R*^2^ = 0.15, with Language group, *p* = 0.88, and the interaction between Language and Age group, *p* = 0.17, not being significant, and Age group being significant, *p <* 0.01.

The Aggregate score was significant, *F*(3, 52) = 5.3, *p <* 0.01, *R*^2^ = 0.23, with Language group, *p* = 0.16, and the interaction between Language and Age group, *p* = 0.22, not being significant, and Age group being significant, *p <* 0.001.

### 4.3 Research question four: episode complexity

A linear mixed effects model was performed with Language group, Age group, Episode number (one, two, three), and the interactions between Language group with Episode number and Age group with Episode number as fixed effects, and Subject as a random effect (see Table 9). Language group was nonsignificant, *F*(1, 53) = 0.69, *p* = 0.41, Episode number was significant, *F*(2, 106) = 11.71, *p <* 0.001, the interaction between Language group and Episode number was nonsignificant, *F*(2, 106) = 1.73, *p* = 0.18, Age group was significant, *F*(1, 53) = 5.5, *p <* 0.05, and the interaction between Age group and Episode number was not significant, *F*(2, 106) = 0.39, *p* = 0.68. Tukey HSD post-hoc analysis revealed significant differences between episodes one and two, *p <* 0.001, and episodes one and three, *p <* 0.05, but a nonsignificant difference between episodes two and three, *p* = 0.08. Episode one had a mean complexity score of 2.04 (*SD* = = 0.54), episode two had a mean complexity score of 1.52 (*SD* = 0.63), and episode three had a mean complexity score of 1.75 (*SD* = 0.72). In sum, the school-age children included significantly more GAO elements than the preschoolers, but there was no difference between the Language and Age groups in which of the three episodes they tended to retell with more GAO elements – all groups retold episode one with the most complexity, followed by episode three, then episode two.

**Table 9:** Linear mixed effects results for language group, age group, and episode number.

Variable	*df*	*F*
Language	1, 53	0.69
Age	1, 53	5.5*
Episode number	2, 106	11.71***
Language*Episode number	2, 106	1.73
Age*Episode number	2, 106	0.39

^+^*p <* 0.1, ^∗^*p <* 0.05, ^∗∗^*p <* 0.01, ^∗∗∗^*p <* 0.001.

## 5 Discussion

The current study examined shared and divergent patterns in the English narrative micro and macrostructure of Japanese-English bilingual and English monolingual children, building on prior research examining bilingual narrative development (Bohnacker et al., 2022; Chan et al., 2023; Gagarina, 2016; Gutierrez-Clellen, 2002; Hao et al., 2019; Iluz-Cohen and Walters, 2012; Kubota et al., 2022; Kunnari et al., 2016; Lipner et al., 2024; Maviş et al., 2016; Otwinowska et al., 2020; Roch et al., 2016; Rodina, 2017). Specifically, microstructure elements – MLUm, NDW, SI, and number of fragments – macrostructure elements – SS, SC, IST – and story comprehension were compared to examine the effects of how age and learning a typologically distinct HL may impact narrative development in the ML. We recruited 56 children (24 girls) ages three to eight (M = 73.6 months, SD = 20.1 months), attending public school in English. Half of the group were Japanese-English bilingual children, the other half were age-matched English monolingual children. The MAIN was used to elicit narratives to analyze the two groups’ micro- and macrostructure, and a caregiver questionnaire was used to gather demographic and language input information. Key findings include: (1) All microstructure and macrostructure measures differed significantly between age groups, except SC; (2) the only microstructure measure that differed significantly between language groups was NDW, with the monolinguals significantly outperforming the bilinguals; (3) there was a significant and nearing significant interactions between Language and Age group for MLUm, NDW, SI and fragments, showing that the bilingual preschool group had the lowest MLUm, NDW, and SI scores, and the bilingual school-age group had the highest number of fragments; (4) no macrostructure measure was significantly different between Language groups; (5) Age and Language groups did not differ in which of the three episodes of the MAIN they tended to retell with greater complexity; specifically, both age and language groups retold episode one with the greatest complexity, followed by episode three, and lastly episode two with the least complexity.

### 5.1 Differences in microstructure

School-age children showed greater syntactic complexity than preschoolers, with higher MLUm and SI scores reflecting increased use of subordinate clauses, verbal morphology, and modifiers. This aligns with research on age-related narrative development (e.g., Justice et al., 2006; Košutar et al., 2022; Roch et al., 2016). School-age children also had higher NDW scores, while bilingual preschoolers showed significantly lower NDW than the other groups, consistent with findings that bilingual vocabulary growth is slower than monolinguals’ (e.g., Bialystok et al., 2010). Within narrative contexts, Tsimpli et al. (2016) reported greater lexical diversity in monolingual SLI children than bilingual SLI peers, and Otwinowska et al. (2020) found monolinguals outperformed bilinguals on type–token ratio. However, in the current study, NDW differences between bilingual and monolingual school-age children disappeared. This may reflect bilinguals’ increasing translation equivalents with age (Kan and Kohnert, 2005; Sheng et al., 2011) or easier acquisition of the more dominant ML compared to the HL (Gathercole and Thomas, 2009). Bilinguals may also take longer to develop English CALP because their input is divided across two languages (Cummins, 1979b). Thus, while bilingual preschoolers had the lowest NDW, SI, and MLUm, bilingual school-age children performed similarly to monolinguals, suggesting that microstructure—and CALP—converges with sufficient language-specific experience.

Input amount may explain this convergence. Cattani et al. (2014) suggest bilinguals need about 60% input in a language to perform comparably to monolinguals. In this study, bilinguals received roughly 55% of at-home input in English; when school input (≈35 h/week) is included, bilinguals received about 43.6 h of English per week versus monolinguals’ 51.5 h—approximately 84% of monolingual input. Thus, the bilinguals exceeded the 60% benchmark, which may have contributed to their comparable school-age microstructure outcomes.

An interesting finding in the current study was that the school-age bilinguals used the greatest number of fragments. This finding parallels the findings of Mishina-Mori et al. (2024). They found significantly higher usage of pronouns and fewer null forms among the bilingual children’s Japanese narratives compared with the Japanese monolingual group. They hypothesized that the excessive use of pronouns among the bilingual children could be interpreted as cross-linguistic influence from English. In the current study, the direction of influence was the opposite, showing evidence of the Japanese-English bilinguals dropping pronouns more often in successive clauses in the English narratives, a potential influence from Japanese. A similar findings was observed by Otwinowska et al. (2020), who found that the bilinguals overused pronouns in Polish, which may have been transfer from English. Overall, while the school-age Japanese-English bilinguals in our study did use more fragments in their narratives than the other groups, this difference was nearing significance and should be further investigated in future studies.

### 5.2 Differences in macrostructure

Much of the research on narrative development posits that microstructure is relatively language-experience dependent, which was reflected in the subtle microstructure differences between the language and age groups. In contrast, macrostructure is generally less tied to language-specific experience (Andreou and Lemoni, 2020; Boerma et al., 2016; Gagarina et al., 2016; Kubota et al., 2022; Rezzonico et al., 2016; Rodina, 2017), though cultural storytelling conventions can influence stylistic features (Fiestas and Peña, 2004; Gorman et al., 2011). In the present study, bilingual and monolingual children did not differ in SS. This contrasts with Kunnari et al. (2016), who found higher SS scores in Finnish monolinguals than Finnish–Swedish bilinguals; their bilinguals were more balanced, possibly receiving more equal input across both languages compared to the English-dominant bilinguals in our study. Both groups most frequently produced SS components such as IST, Initiating Event, Attempt, and Outcome, while Setting and Goal were least frequent—similar to the frequencies of Rodina (2017), and Bohnacker (2016), who also reported infrequent Goal statements. Setting information was rare despite its importance (van Dijk, 1976b); only one child provided both time and location, echoing Bohnacker’s (2016) finding that scene-setting occurs in only 25% of 5-year-olds’ narratives and 61% of 6–7-year-olds’. It may be the case that only some of the children took on a “narrative mode,” and used stereotypical phrases like “once upon a time,” or “the end,” which may have elicited more expositional or scene-setting information, whereas other children used more of a “picture-description mode” (Montanari, 2004) and focused on recitation of the events. Overall, our findings suggest that story structure is relatively language-general: SS knowledge does not appear affected by reduced input and may transfer across languages.

In contrast to the findings on SS, SC showed less of a consistent developmental trend with age for both groups, and was only predicted by grammaticality and lexical diversity in the bilingual group. SC has been operationalized in a variety of ways (Lindgren et al., 2023). In the current study, we employed a 3-point system (Chan et al., 2023; Maviş et al., 2016), which is different than the recommended scoring system of the MAIN manual, which employs Westby’s decision tree (Westby, 2012). Therefore, the inconsistent findings in SC may be explained by the SC scoring system used in the current study. It is also noted that the current study used MAIN only as a story retell, rather than as a story tell (e.g., Kunnari and Välimaa, 2020; Maviş et al., 2016), which may have impacted the outcome. However, our findings are similar to Chan et al., 2023, who employed the same SC scoring system. They found that SC was predicted by both NDW and grammatical skills. On the other hand, Maviş et al. (2016), who also employed the same SC scoring system, found that SC was correlated with age, whereas, in the current study, SC was not correlated with age for either group. In contrast to our study, Maviş et al. used the MAIN as both a story tell and story retell task. The difference in task demands may explain the differences in findings. However, a lot of the research using MAIN has found that, regardless of how SC was scored, children tend to rely on Attempts and Outcomes, often omitting Goals (e.g., Bohnacker, 2016; Bohnacker et al., 2022; Gagarina, 2016; Lipner et al., 2024; Rodina, 2017), which was also the case for the current study. It is possible that the SC scoring system of MAIN may not fully capture the process of summarizing the essential “point” of a narrative. In telling or retelling a story, the narrator summarizes the “gist” through operations like deletion (removing redundancy) or integration (combining components) (van Dijk, 1976a). The narrator chooses which information can be omitted because it can be inferred. Since goals can be inferred (Lindgren et al., 2023), it seems logical to omit them for the sake of economy. While MAIN offers a structured and widely used approach to assess narrative macrostructure, the standardized structure potentially might not capture differences in how a child summarizes a story, or culture-specific stylistic conventions in narrative discourse that could vary in more naturalistic language samples.

Notwithstanding the significant difference between the bilingual and monolinguals on NDW, there were no significant differences between the groups on ISTs. This finding is similar to Kunnari et al. (2016), who found no difference between Finnish-Swedish bilinguals and their monolingual peers in number of ISTs. Additionally, ISTs were strongly correlated with NDW in both groups, which is similar to the findings of Altman et al. (2016) and Chan et al. (2023). This finding is interesting because, while the microstructure measure of NDW was different between groups, there were no differences between either group on ISTs, suggesting that use of ISTs is not significantly impacted by differences in lexical diversity.

Story comprehension improved with age, especially on the more difficult ToM questions (Kunnari and Välimaa, 2020; Lynch et al., 2008; Paris and Paris, 2003). However, there were no language group differences in narrative comprehension. The bilingual group had a slight, yet nonsignificant, advantage on the final ToM question. Interestingly, Rodina (2017) observed a similar trend, with the bilinguals scoring higher than monolinguals on the ToM questions, but only in Norwegian, the ML. This finding is in line with research on bilinguals’ advantage in control of processing (Bialystok, 2001). Comprehension question 10 for the Dog and Cat stories asks if the boy and the dog/cat will be friends after the dog/cat has stolen something from the boy. Many of the monolinguals answered along the lines of, “Yes, because the boy might like animals.” The bilinguals tended to answer correctly, that being, “No, because the dog/cat stole from the boy.” The bilingual group may have been able to observe the situation with more control and answer the question with the most logical response, despite other plausible possibilities. Aside from this minor difference, both language groups scored close to the ceiling (see Table 4). It is to be expected that narrative comprehension scores are higher than narrative production scores, as comprehension is a fundamental prerequisite for production (Curenton, 2011); however, the distribution of answers for both groups is negatively skewed according to a Shapiro–Wilk test, *W* = 0.75, *p <* 0.001. In the current study, the mean comprehension score reached 8.6/10 by four years old, and 9.25/10 by five years old, indicating that the MAIN comprehension questions might be too easy for its age range.

Finally, the aggregate MAIN score was strongly correlated with age, vocabulary, and syntactic complexity in both groups. Squires et al. (2014) describe how children first learn a general framework for telling stories; then, as their ability develops, they begin providing more details in their stories through use of more complex and explicit microstructure elements. The children in the current study improved in all aspects of micro and macrostructure with age (except for SC), as they began integrating the two with greater intricacy. The aggregate score had the most robust correlations with age, vocabulary, and syntactic complexity, particularly for the bilingual group, suggesting that it is a strong measure of narrative development in the ML for both monolingual and bilingual children. Coupled with microstructure measures of lexical diversity and syntactic complexity, the aggregate score may provide a comprehension indicator of the child’s narrative skills.

### 5.3 Differences in episodic complexity

The present study found that both groups retell episode one of the MAIN with the most complexity, followed by episode three, and finally episode two with the least complexity; this finding was also consistent between the preschool and school-age children. This finding differs from Lipner et al. (2024), who found that in Hebrew-English bilingual children, episode three of the MAIN yielded the most complexity. This may be due to the different procedures. Lipner et al. (2024) had children retell the story to a puppet who “was sleeping” while the examiner was telling the story. This was to control for the effect of shared knowledge, which is controlled for in a different manner in the MAIN manual (see “methods”). This manner of story retell elicitation could have changed how the participants identified important or unimportant information. Additionally, Lipner et al. (2024) explained their finding saying that, in the third episode, all the characters meet, and relationships become explicit, making it easier to relate to the characters and “finalize” the story. In our study, it is possible that the children added more details to the first and third episodes, given their perceptual salience. The second episode is amid the end of the first and beginning of the third, which may have made it more difficult to remember and recall (see Appendix for examples).

### 5.4 Limitations

As with any study, there are many limitations. The absence of significant effects for the macrostructure measures may partly reflect limitations in sample size and composition. Although the number of participants was consistent with comparable research on bilingual and monolingual narratives (e.g., Kunnari et al., 2016; Rodina, 2017), the relatively small sample and broad age range may have introduced developmental variability, making it more difficult to group the bilinguals by both English and Japanese proficiency, and reducing the sensitivity to detect more specific group or age effects. Additionally, incorporating other measures, such as phonological, memory, or vocabulary measures would have provided a more detailed picture of both groups. While the MAIN does provide a thorough analysis of a child’s language, additional measures can help further explain the results found by the MAIN. We also would have liked to query more about SES and literacy skills within the caregiver questionnaire to fully round out the findings from the MAIN. Finally, the bilingual group used in the current study was English-dominant, and they were not grouped based on Japanese proficiency. Therefore, our findings on cross-linguistic transfer should be interpreted with caution, particularly for bilingual children with more homogenous profiles of language ability in both languages, or more Japanese-dominant profiles. More research with Japanese-English bilingual children who are more balanced or more Japanese-dominant would add to the body of research on bilingual narrative development, as well as using the MAIN as an effective clinical and research tool. Future directions would include examining Japanese-English bilinguals longitudinally or examine both languages of the bilingual children to further explain how multilinguals ad monolinguals differ and overlap in narrative development.

### 5.5 Clinical implications

This study has important implications for researchers and SLPs. The MAIN script used in this study was adapted into Japanese using the recommendations of Bohnacker and Gagarina (2020). This adapted script can further the theoretical development and clinical utility of MAIN by broadening the populations that researchers can use it with (Kan et al., 2020). For SLPs, language sample analysis is a comprehensive and unbiased way of evaluating the languages of bilingual children (Botting, 2002), and narratives are a good index of development over time (Liles, 1993). This study shows that macrostructure as measured by the MAIN – specifically the aggregate score – emerged as a potential index of language development. Additionally, this study elucidates what might be expected from the narrative of a typically developing Japanese-English bilingual child, that being, potentially lower lexical diversity and syntactic complexity, and more frequently omitted subject pronouns. Gutierrez-Clellen et al. (2000) point out that measures used to assess children’s language are typically only useful for monolingual English speakers (e.g., word order, omitted pronouns, etc.). Therefore, when assessing Japanese-English bilingual children, certain deviations in grammar and vocabulary ought to be expected. Ultimately, it is essential that the SLP be knowledgeable about the basic characteristics of the languages that their students speak to appropriately tease apart a difference from a disorder.

## Data Availability

The raw data supporting the conclusions of this article will be made available by the authors, without undue reservation.

## Appendix

**Structural complexity examples**

**Episode 1:**

- GAO
  So, one day a cat saw a butterfly on the bush.
  And the cat wanted to catch the butterfly. (Goal)
  The cat leaped over the bush to try to catch the butterfly. (Attempt)
  But he fell into the bushes and hurt himself. (Outcome)
- AO
  The mouse was sitting there.
  He’s going to chase it. (Attempt)
  He was running when he bumped into the tree. (Outcome)
- O
  There was a playful dog.
  The mouse ran away very quickly.
  The dog bumped his head. (Outcome)

**Episode 2:**

- GAO
  The boy was so surprised to see the cat in the bushes that he dropped the ball.
  He was sad that the ball went into the water.
  He wanted to get it back. (Goal)
  The boy tried to get the ball back. (Attempt)
  He got it and was very happy to have his ball back. (Outcome)
- GO
  And while the boy was thinking how he wanted the ball, the cat saw the bucket. (Goal).
  And the boy had the ball back. (Outcome)
- A
  The boy is carrying a bag with sausages.
  His balloon slipped out of his hands.
  He’s getting the balloon. (Attempt)

**Episode 3:**

- GAO
  The cat saw a bucket of fish.
  And wanted to eat one. (Goal)
  The cat went to get the fish from the bucket. (Attempt)
  And the cat was happy to get fish. (Outcome)
- GA
  And the dog noticed the sausages.
  And he said he wanted to grab them. (Goal)
  And the boy did not notice the dog was trying to grab them. (Attempt)
- O
  And the dog saw the sausages.
  And the man was so happy that he did not notice that the dog ate his sausage. (Outcome)
